# Hepatocellular carcinoma with subcutaneous metastasis of the scalp

**DOI:** 10.2478/v10019-011-0022-7

**Published:** 2011-07-20

**Authors:** Yilmaz Tezcan, Mehmet Koc

**Affiliations:** Selcuk University, Meram Faculty of Medicine, Department of Radiation Oncology, Konya, Turkey

**Keywords:** hepatocellular carcinoma, radiation therapy, cutaneous metastases

## Abstract

**Background:**

The majority of subcutaneous metastases from hepatocellular carcinoma (HCC) originate from needle tracks or surgical wound contamination. Non-iatrogenic subcutaneous metastasis from hepatocellular carcinoma was rarely reported.

**Case report:**

A 70-year-old man presented with a mass in his left occipital region of the scalp. The surgical complete resection was performed. The histopathology report of the scalp mass showed a characteristic metastatic HCC. Computed tomography (CT) of the abdomen showed no primary or metastatic lesion in the abdomen; that’s why the adjuvant treatment was not given after the surgery. Five months later, magnetic resonance imaging (MRI) of the brain revealed a 6 × 5.5 cm mass at the left posterior parietal region of the scalp. Second surgery was performed and histopathology of the specimen excised was again metastatic HCC. The external beam radiation therapy (XRT) was administered after the surgery. A follow-up MRI of the brain showed no recurrent disease after 9 months from XRT.

**Conclusions:**

HCCs should be considered in the differential diagnosis of carcinomas metastatic to the skin, even in the absence of liver symptoms.

## Introduction

Hepatocellular carcinoma (HCC) is the most common primary tumour of the liver. Lungs, abdominal lymph nodes, and bones are the most common extrahepatic metastatic sites of HCC.

Cutaneous metastases from HCC are very rare.^1^–^2^ We report a case who has a subcutaneous mass on his scalp which was the first clue for the diagnosis of the HCC. Aggressive recurrence was occurred three months after surgery that was well controlled with radiation therapy.

## Case report

**FIGURE 1:**
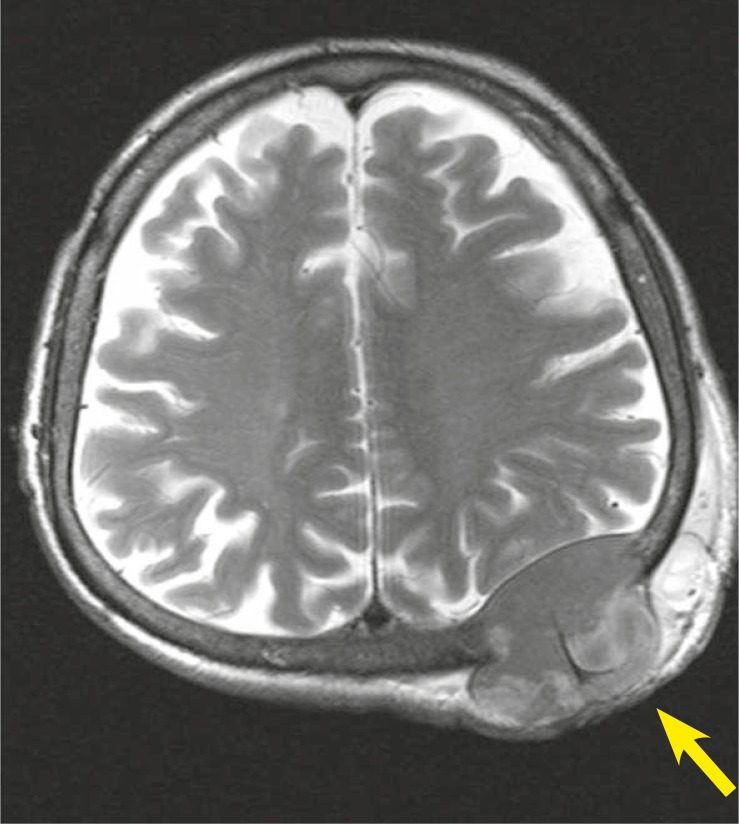
Brain MRI with a subcutaneous metastasis from hepatocellular carcinoma.

In November 2009, a 70-year-old man presented with a mass in his left occipital region of the scalp. His ECOG status was 0. He has a history of hepatitis-C virus (HCV) positivity for 30 years. The magnetic resonance imaging (MRI) revealed a 6.5 × 6.0 cm mass invading bone in the left occipital region of the scalp which has extra and intracranial components (Figure 1). Fine needle aspiration biopsy showed a malign tumour.

The mass was completely resected. The macroscopic size of mass was measured 6.0 × 5.5 × 2.0 cm. Pathology of the mass showed a characteristic metastatic hepatocellular carcinoma (HCC) invading the occipital bone. All surgical margins were free of tumour. Immunohistochemical staining showed Pan-CK, CK8, CEA(p) and CD10 positivity.

**FIGURE 2:**
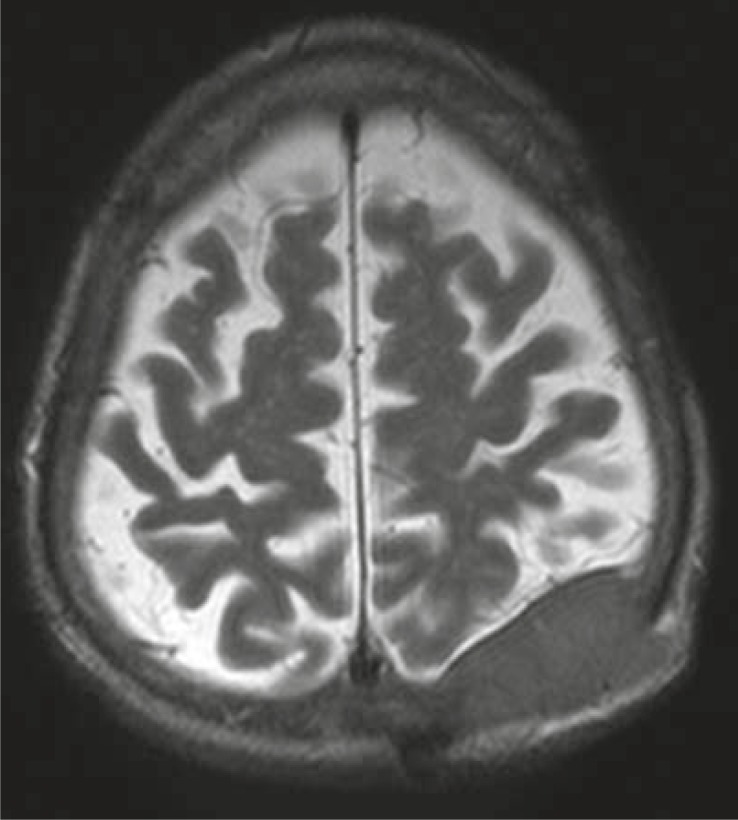
Brain MRI with a recurrent subcutaneous metastasis from hepatocellular carcinoma.

**FIGURE 3:**
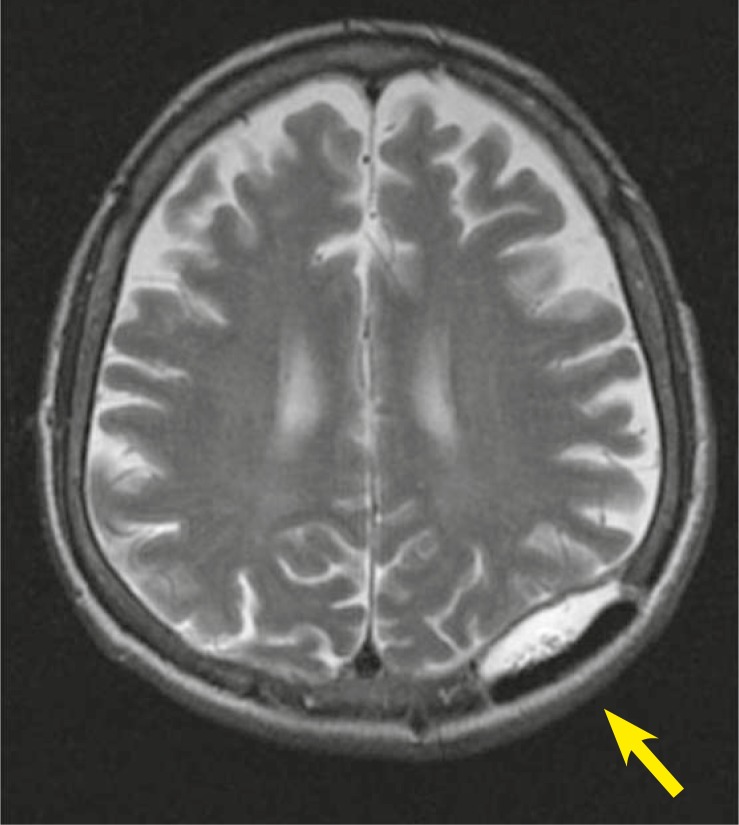
Brain MRI after second surgery which is contrast enhanced residual lesion.

**FIGURE 4:**
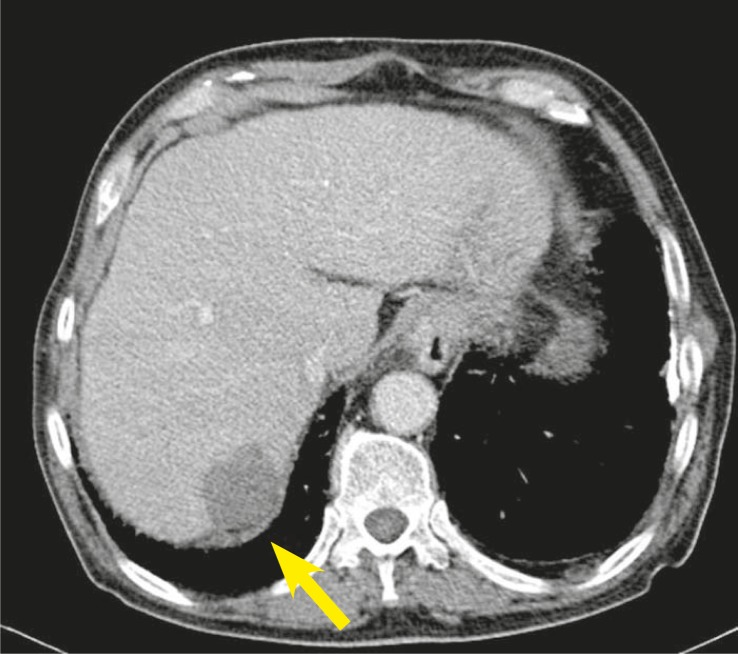
CT scan of hepatocellular carcinoma in right lobe of the liver.

**FIGURE 5:**
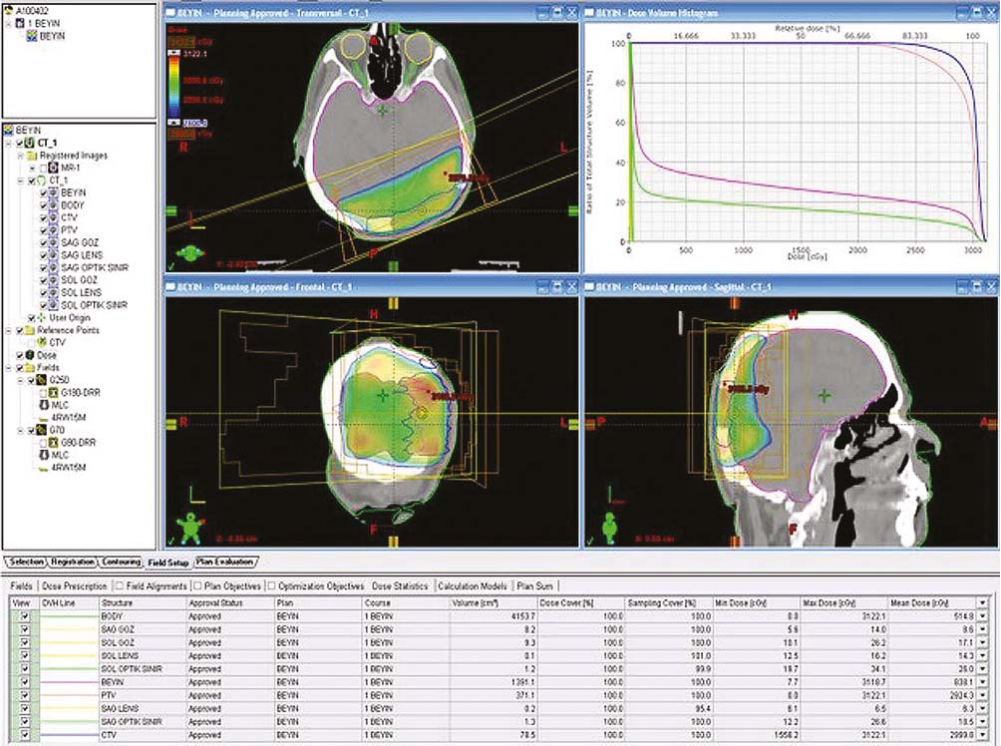
The typical dose distribution from 2 posterior oblique field using 6 MV photon beams from conformal radiotherapy plan.

**FIGURE 6:**
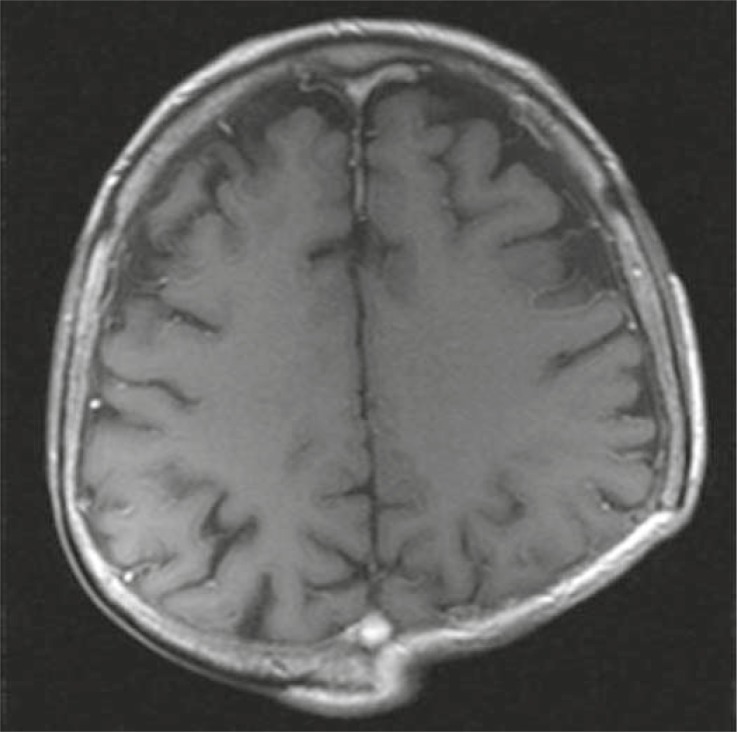
MRI of the brain 9-month later of the XRT.

Complete blood count, liver function tests, and α-fetoprotein (AFP) level were normal. Abdominopelvic computed tomography (CT) showed no abnormalities; that’s why no adjuvant treatment was given after the surgery. Five months later (on April 2010) he noticed a mass at the same region. MRI revealed a 5.2 × 2.3 cm (Figure 2) mass invading bone in his left posterior parietal portion of the scalp. For the second time the surgery was performed and once again the metastasis of HCC was confirmed on the histopathological examination of the resected tissue specimen. Postoperative MRI of the brain revealed a contrast enhancing mass on the left parietal region (Figure 3). CT of the abdomen showed a hypodens lesion in the right lobe of the liver (Figure 4). Due to the bone invasion and residual disease, the palliative external beam radiation therapy (XRT) was applied after the surgery. Three-dimensional treatment planning was used and the radiation dose to the scalp was 300 cGy per day for 5 days a week; the total dose was 3000 cGy (Figure 5). Radiofrequency ablation (RFA) was administered to the metastatic mass of the right liver and the systemic therapy with a targeting agent (sorafenib) treatment was started. A follow-up MRI of the brain showed no recurrent disease 9 months from XRT (Figure 6). After 17-month follow-up from the diagnosis, the patient could perform his daily activities although he developed hypo albuminemia and fatigue.

## Discussion

The majority of subcutaneous metastasis from HCC originates from needle tracks or the surgical wound contamination.^3^–^5^ The non-iatrogenic subcutaneous metastasis from hepatocellular carcinoma was rarely reported. Since these patients are usually considered at their terminal period, they are usually observed without any treatment. However, the surgical resection of the metastatic lesion has been performed in a few cases.^5^,^6^ In one study, skin metastases were detected in only 2.7 % of cirrhotic HCCs, and none in noncirrhotic HCC.^7^ Although the subcutaneous metastasis of HCC is unusual, it could be presented as the sole and initial sign of the HCC.^8^

Huang *et al*.^9^ reported that the radiation therapy was found XRT an efficient treatment modality when subcutaneous metastases of HCC are in question. They observed at least a partial response in 20 of 24 lesions (83.3%), with radiation doses ranging from 8 to 64 Gy. No severe squeals were recorded. The overall 6-month survival was 43.4%, and the overall 1-year survival was 22.8%. At these patients, the treatment response was good, and the side-effect profile was acceptable. Due to our palliative aim, we applied 30 Gy in 10 fraction XRT over 2 weeks and achieved a good local control during last 9-month.

HCCs should be considered in the differential diagnosis of carcinomas metastatic to the skin, even in the absence of liver symptoms or absence of imaging finding with ultrasonography or CT that usually reveal the primary lesion.^10^ Surgery is the primary treatment choice, like in some other cases of metastases^11^, in particular, when superficial (skin) metastases are to be resected. Radiotherapy seems to be a reasonable alternative in patients with advanced disease and poor performance status and in other clinical scenarios when surgery could not be implemented.
